# Remarks, Gleanings, and Extracts

**Published:** 1874-06

**Authors:** S. M. Burnett

**Affiliations:** Tennessee


### Remarks, Gleanings and Extracts.

BY S. M. BURNETT, M.D., OF TENNESSEE.

*Physical Traits of Epileptics.*—In the London *Medical Record* for May 13th is an admirable notice of Sanders' paper on the "Medico-legal Aspects of Epilepsy," from which we extract the following:

"When the fits are very numerous, it is common to say that the epilepsy is the *cause* of the insanity. Of course both are symptoms of the common disease which underlies them, and are mutually equivalent. Yet doubtless the fits themselves are prejudical. The school dogma that epilepsy may, but need not necessarily, have mental unsoundness as its sequel may be true to some extent; but the answer would probably take a different form if both the epilepsy and the mental disturbance were regarded as collateral symptoms of a brain lesion of a serious nature. The physical characters of epileptics are well known to be characterized by mental weakness—which may range from perfect imbecility to these slighter degrees of impaired intellect—which can only be recognized by close and careful observation, and then, perhaps, with difficulty. The persons who give us difficulty, in a forensic point of view, are those whose mental weakness is but slight; who conform themselves, in most things, to the ordinary customs of society, and whose peculiarities are not obvious except to their intimates. But it may help us to remember that, however slight the intellectual failure, epileptics nearly always exhibit abnormalities of feeling. Their irritability is proverbial, and it leads them to abnormal actions and reprisals against real or fancied encroachments. Hot temper, rash anger, and, when it comes to action, brutal want of reflexion, characterize epileptics under all circumstances of life, whether trifles or more mighty matters are at stake. Besides intense irritability, they often manifest a blending of the finer feelings, which leads to an almost total disregard of the ordinary feelings of decency and propriety. Epileptics are not bound by the tie of relationship, friendship or love, like other people. Most of them are egotistic to the last degree. In many of them, particularly females, there is, along with mental weakness, a peculiar childish and clinging manner, which may afterwards become erotic. This is deceptive to the uninitiated, and often conceals unreasoning hate. Epileptics can manage some occupations very well. But only the minority really succeed in life; the great majority suffer shipwreck, if not at school, in the factory, or in the counting-house, etc. Epileptics are also prone to all sorts of crooked dealings and perverted activities, and exhibit but little power of resistance against temptations of any kind. He is also of the opinion that "moral insanity" must often be due to masked or larval epilepsy.

*Commotio Retinæ.*—Prof. W. W. Seely reports two cases of this affection treated by galvanism successfully, in the *Clinic* of April 25th. In both cases there had been blows on the eye--one five days and the other three days before Dr. Seely saw them. No abnormality was found on ophthalmoscopic examination; nevertheless, there was almost complete extinction of the sense of vision. The zinc pole of a battery of ten cups was applied to the neck, and the other pole was passed over the closed lid for a minute. A single application was followed by restoration of vision, and, after a second application, it was increased to the normal standard.

In connection with the above, we will quote Prof. Arlt's remarks on the subject of commotio retinæ, to be found in his paper on "Injuries to the eye in their medico-legal relations," in some recent numbers of the *Wein. Med. Wochenschr.*:

"Not only before, but also after the introduction of the ophthalmoscope into practice, it was usual, even in recent times, in cases of disturbance of vision after injury, where no changes could be detected by unaided vision or by the ophthalmoscope, to locate the lesion within the skull, to use the term *commotio retinæ* in explanation of the partly transient, partly permanent disturbance of vision, and to assume a minimum degree of disturbance of the elements of the retina, or a paralysis of the vaso-motor nerves through concussion. Dr. Berlin pointed out the untenability of this supposition, and struck out the path of more exact observation.

"We may with more probability assume that concussion of the retina has taken place, when the eye alone has been struck with a blunt instrument. This group is divided into two groups: those in which disturbance of vision—generally of a high degree, and accompanied with impairment of excentric vision—is permanent, or at least of long duration; and those which show only a slight impairment—*i. e.,* of central vision alone, which generally passes off in a few days. From repeated observations, I agree with the opinion of Geissler and Knapp, that amaurosis or a high degree of disturbance of vision after concussion of the eye very rarely occurs without changes which may be discovered by the ophthalmoscope.

"As regards the explanation of the milder cases by assuming the thrusting of the elements of the tissues against each other, this is just as little tenable with regard to the retina as to the brain; and with regard to the hypothesis of paralysis of the vaso-motor powers, Berlin could not, after careful comparison of the injured with the sound eye (in man and in rabbits), discover any dilatation of the arteries or veins. From his observations and experiments, Berlin arrived at the conclusion that in all cases where concussion of the eye is followed by slight disturbance of central vision, or unequal impairment of excentric vision, and recovery follows in a short time, the case has been merely one in which there has previously been irregular astigmatism. This assumption is partly strengthened by the constant resistance of the iris to atropia, partly by the anatomical changes found in experiments on rabbits in the anterior part of the eye.

"In experiments upon rabbits it was found, on examination with the ophthalmoscope, that when the injury had fallen on the sclerotic, extensive opacity was found on the part of the retina corresponding with the part of the sclerotic that was struck, and also a similar opacity in a neighboring part of the fundus oculi lying directly opposite the struck part. On anatomical investigation, the important discovery was made, that the retinal opacity was only due to acute œdema, dependent on hemorrhage between the choroid and sclerotic. Thus we have here to deal essentially with a rupture of the choroidal vessels."

*Signs of Death.*—There is nothing more horrible than the thought of being buried alive. Indeed, this constitutes one of the strongest objections to the present system of inhumation, and will do more, perhaps, than any other one thing to advance the plan of cremation of the dead, which is now exciting so much interest in so many different parts of the world.

The Marquis d'Ourches left to the Academy of Sciences, in Paris, a prize of 20,000 francs, to be awarded to any one who would discover a simple and popular means of recognizing the signs of real death, which may be put in practice by the poor and uneducated villages. He also left another prize of 5,000 francs for the discovery of a scientific method of recognizing with certainty the signs of actual death. One hundred and two works were sent, but of all none were entitled to the first prize. The second prize of 5,000 francs has been divided between four individuals, each of whom has presented a scientific mode that is infallible in recognizing the presence of actual death; and, in addition, several modes were found deserving of "honorable mention." One sign, for which 500 francs was awarded, was the modification presented by burning the pulp of the finger in a flame. The blisters following this application, if the body be dead, will be filled only with vapor; whereas, if it be living, the contents will be serous. Another method, which received "honorable mention," is founded on the fact that, after death, circulation in the capillaries is stopped. Consequently, if a cupping glass is applied some time after death to the pit of the stomach, aud the surface afterwards scarified, it will give out no blood. Valuable and certain signs of death are also obtained from the eye. The fundus of the eye, when viewed with the ophtholmoscope, will show in death a general discoloration, instead of the bright red reflex we see in life. Another sign, applicable by those not expert in ophtholmoscopy, is a gray and cloudy spot first appearing on the external portion of the sclerotic, and which finally takes entire possession of it. Some hours after death the muscles lose their power of responding to the electric current, and this law can be brought to bear in the determination of actual death. Another sign, which does not require any scientific knowledge for its determination, is the presence of violet-colored spots in the dependent parts of the body, which make their appearance very shortly after death. In 15,000 cases examined, it was not absent in one. For these investigations the sum of 2,000 francs was awarded. The old theory of the hydropathic Thompson that "heat was life and cold death" is not without a foundation in truth. It is a certainty that life cannot exist below 68° Fahr.; but as the body, even after death, will still remain of the same temperature of the surrounding atmosphere, and, as this is often above 68°, it cannot always be depended upon. Consequently this should be relied upon less than the gradual and progressive cooling which follows death. It has been determined that the body after death loses heat at the rate of of 2° cent. every hour. Death can thus easily be determined by the application of the thermometer every hour after apparent death. Another plan recommended is the application of the stethoscope to the cardiac region. If the heart is heard beating ever so faintly life is not extinct; but if, after a prolonged examination, no cardiac sounds are heard, life's pendulum may be deemed to have stopped its oscillations.

*What Food is most Digestible?*—In the treatment of gastric disorders it is of first importance that the food taken into the stomach should at the same time be nutritious and easily digestible. Prof. Leube, of Jena, in his Treatise on diseases of the stomach, makes the following observations on this point:

"Of the different kinds of meat, young veal, fowl and pigeons are digested with least difficulty. With many persons, fish and beef must be placed in the same catagory; and it is advised to give the former in the boiled state only, while beef is recommended to be brought on the table roasted, but retaining a red color. It is an old culinary observation that excessive roasting and boiling both render meat tough ; and, in accordance with the latest published researches of Frick, have shown that the same digestive juice requires three times as long to act on boiled meat as on raw. In the use of roast meat, it is an established rule, even among the non-professional public, to abstain from the addition of fatty sauces—a rule that finds its justification in the fact that pieces of meat enveloped in fat are less readily acted on by the gastric juice than are peices of lean meat. The experience of thousands of years has proved milk and eggs to belong to the class of easily digestible substances. Milk is naturally borne well by many persons suffering from gastric disease, as all must have observed ; and raw eggs are said to give but little trouble to the stomach. This latter statement, however, is not confirmed by Dr. Leube, who has found boiled eggs more digestible than raw ; and Frick's most recent observations show also that raw albumen has no advantage as regards digestibility. Dr. Leube allows his patients to use only soft boiled eggs. Of vegetables, the most tender only are to be allowed, such as asparagus, young hops, shelled sugar peas and young carrots. Potatoes should never be eaten except in the form of *pureé.* Gruel containing particles of groats, which may irritate the stomach, is specially to be avoided. The bread should be wheaten, and absorbs the gastric juice better if it have been baked for some time. Alcoholic liquors are to be avoided as much as possible. If wine be urgently desired, it may be given equally well *per anum* as by the mouth.—*London Medical Record.*

*Influence of Alcohol on the Temperature of the Human Body.*—The following general results of some most carefully conducted experiments by Rilgel, on this subject, we take from the London *Medical Record.* They were originally published in the *Deutches Archivo fur Klin, Med.:*

1. Alcohol depresses the temperature, not only in febrile diseases, such as typhus, erysipelas and pneumonia, but in a febrile condition also. This depression generally amounts to only a few tenths of a degree (cent), and lasts for a short time only. Very rarely there is an equally great rise of temperature. 2. In those recovering from severe illness, the downfall is somewhat less, or, more often, altogether absent. This is also the case in those habituated to alcohol. 3. The larger the dose the greater the downfall of temperature. 4. Riegel concludes that, although alcohol scarcely deserves the reputation given to it in England, as a decided depressor of temperature, yet, on the other hand, it never essentially raises the temperature—the constant dread of centinental practitioners—and it is decidedly one of those things which diminish bodily waste, like tea and coffee.

"CASTE, the celebrated naturalist, was almost sightless at the time of his death, having destroyed his vision with the microscope."

We quote the above paragraph from one of our exchanges, and with all due deference to it, and the source from which it obtained its authority for the assertion, we feel compelled to say that we can't believe a word of it. There is nothing more in the use of the microscope that is calculated to injure vision, than in the various offices the eyes are called upon to perform in the ordinary duties of every-day life. The accommodation is not put to any severe tension, because the instrument can be adjusted to any required degree of accommodation. The brightness of the illumination can not be injurious, for a light of such brightness as to be dazzling is just what we *don't* want in making microscopical examinations. The position of the head when the microscope is erect might have an evil influence if the patient is myopic, or where a slight congestion of the eye is to be avoided; but the stands made now, that can be inclined to any angle, obviate that difficulty entirely.

A close and accurate examination of any object for a long time is fatiguing, but the fatigue is more mental than ocular. We know gentlemen who are myopic that have for many years used the microscope daily, and often for hours at a time, and have never experienced any inconvenience to their vision thereby.

*Regeneration of the Crystalline Lens in some Mammals.*—M. Millot (*Journal de l'Anat et Phys.*, 1872), in his elaborate essay on the regeneration of the lens in some mammals, shows that this certainly can occur, the tubes following in the mode in which they are reproduced, the same order that they present during their generation and embryonic development. The regeneration can only occur in the cavity of the capsule, and is in direct relation with the thickness of the cortical layers that are left, especially in the equatorial part, whilst it is in inverse proportion to the age of the animals and the amount of lesion to the capsule. The posterior crystalloid does not take part in the process of regeneration. The first traces of regeneration appear about the end of the second week after the operation, and it is not complete until some time between the fifth and twelfth month, or even later when the animals are old. The new lens never exceeds one-half the size of the natural or old one, (Leroy d'Etialles and some others have obtained them of full size.) The form and size of the new lens resembles that of the old, and it can replace the latter physiologically. The lens can even be to some extent replaced a second time. The microscopical structure of the elements resembles that of the normal organ.

*Spontaneous Retinal Pulsation as a Symptom in Basedows Disease.*—Dr. Becker considers this in a article in the *Annal o'ocul,* translated for the *Detroit Review,* by Dr. J. F. Noyes:

The essential cause of the phenomenon he conceives to be a paralysis of the vaso-motor nerves distributed to the vessels of the retina and the increased action of the heart. The paralysis of the vaso-motor system he considers to be the prime cause of the goitre and exophthalmus; the paralysis of the muscular walls of the thyroidal and post-ocular vessels allowing a much larger influx of blood than normal, while the outflowing is not proportionately increased. From his investigations he cannot conclude that the sole origin of the disease is a lesion of the sympathetic nervous system, as has been thought by some. It is pretty certain, however, that the disease must take rank among the neuroses. Slight inflammation of the iris and corpus ciliare exercises no harmful influence over the process of regeneration. On the contrary, it seems to aid it; panophthalmitis of course prevents it. When neoplastic connective tissue is formed in the cavity of the crystalloid it is derived from exudation poured forth by the iris or vitreous humor.—*Med. Chir. Review.*

*Remarks on Embolism.*—Under this head, Dr. Loring, of New York, discusses, in the *Journal of Medical Sciences* for April, the question of the existence or non-existence of embolism of the retinal artery in those cases of sudden blindness which have hitherto been deemed due to this cause. He gives three cases, and the result of the macro and microscopical examination of one eye that was exhibited. From the result of this examination, as well as the ophthalmoscopical examination and the clinical histories of the cases, he is forced to the conclusion that, at least in these cases which he reports, not embolism, but thrombosis, is the pathological condition. In the eye examined, no other pathological lesion of any importance was found, except a thrombosis of the large choroidal veins.

He accounts for all the phenomena connected with these cases of so-called embolism, according to his theory of thrombosis, and in a manner more satisfactory, in his opinion, than can be done on the theory of embolism. A careful reading of the article has convinced us that there is force and point in his opinion. Still, we have much to learn respecting this very remarkable affection, and we hope much from the investigation of such men as Dr. Loring.

THE administration of eucalyptus as a febirfuge has not been found so successful as it was hoped. It certainly cannot supplant quinine.

*Physiology of the Secretion of Tears.*—In the *Archiv. fien Oph, Band xix., Abth. 3d,* Dr. Reich gives an article on the physiology ot the secretion of tears. His experiments lead him to the following conclusions:

"That the centrifugal nerve-fibres, which are employed in the innervation of the lachrymal gland are situated above the superior cervical ganglion, and perhaps in the medulla oblongata, where are situated the fibres of the origin of that sympathetic nerve which regulates the salivary secretion. Irritation of other nerves, such as the fourth pair, which anastomose in the cranium with the trigiminus, has not given any result. The functional relation resting between the production of tears and the contraction of certain muscles of the face in weeping, laughing, etc., has led the author of this memoir also to study the action of the facial nerve; and he has established the fact that, after the section of this nerve, the reflex secretion always remains the same."—*London Med. Record.*

*Pathogenesis of Tumors.*—Dr. S. W. Cross gives, in the *American Journal of Medical Sciences,* a sketch of all the prominent theories and hypotheses on this subject that have been advanced in modern times, and renders his judgment as follows:

" . . . I am led to sustain the more conservative views of many distinguished observers and experimenters who teach that all the cells of the organism, whether fixed or mobile, epithelial or connective, investing or secretive, lend their aid in the development of tumors, but with the restriction on my part, that the migratory cell is principally concerned, as in the inflammatory process. In other words, the genesis of morbid growths is characterized by the hyper nutrition and multiplication of the masses of protoplasm influenced by it, which are, as a rule, direct productions of the vascular and connective tissue systems."

*Food for Infants.*—It should be remembered that infants cannot digest starchy matters. Starch must be converted into sugar before it can be assimilated, and the infant has not the power to make this change. Hence, this kind of food should be withheld from those infants that are to be brought up by hand. On this point, Dr. Lankester says, in his "Plain Rules for the Management of Infants":

"Most of the deaths from hand-feeding, under six months old, arise from the use of *corn-flour, arrowroot, baked flour, and other kinds of starchy food,* which contain no nutritious qualities, and can never be used as substitutes for milk without endangering the lives of the children who are stuffed with them. When milk cannot be obtained, pure 'condensed milk' can be used with advantage."

*Sleeping Sickness.*—The *Gaz. Medicale de Paris*, of November 29, 1873, contains a review of a paper presented by Dr. Emanuel F. Ribiers to the *Socidade das Sciencias Medicas da Lisboa*, on a curious disease observed by him in the Island of Principe. The principal symptom, and almost the only one, is an irrisistible desire to sleep. It seemed confined to subjects of African or mixed blood, and, as far as observed, was always fatal. A similar disease is mentioned by Dutrouleau, as indigenous to the coast of Africa; and the English naval surgeon, Davis, stationed at Lisbon, furnished further particulars of its occurrence in Africa and elsewhere, and gave several hypotheses regarding its cause—malaria, and the use of various vegetable products, being among them. Dr. Amado was disposed to consider melanæmia as a cause of the affection.—*Chig. Jour. Nerv. and Ment. Diseases.*

In a recent report on the condition of the English hospital at Pekin, China, the attending physician gives a formula for "anti-opium pills." This remedy is composed of extract of henbane, extract of gentian, camphor, quinine, cayenne pepper, ginger and cinnamon, with castile soap and syrup to form the mass, and licorice powder to form the coating. The efficacy of these pills in overcoming the opium habit, and in preventing the suffering on giving up the use of that poison, is stated to have been proved in numerous cases. The native remedies, it is said, contain opium in some form, and most frequently the ashes of opium already smoked, and consequently are inefficacious—it being as difficult to discontinue the use of the medicine as of the drug itself.

*Indigestion.*—After a very prolonged investigation and experimentation on the subject, Prof. Leube has arrived at the conclusion that in indigestion there is more frequently a deficiency of the digestive acid (generally conceded to be hydrochloric) than a disproportion of any other constituent of the gastric juice. He has rarely found such a deficiency of pepsin as would cause any material impairment of digestion. When he finds such a deficiency of acid he gives eight drops of hydrochloric acid in wineglass of water, an hour after taking food, and in some cases repeats the dose four hours afterwards.

Prof. Haughton has found, by direct experiment, that the muscles of women, though inferior to those of men, in exertion for a short time are more capable of long-continued work. The reason he assigns is the smaller muscular fibres of the former, whereby a larger amount of blood is sent to them than to the larger fibres of men; for the smaller the fibres of a muscle the larger the amount of blood sent to it.

Dr. Turner, in the *Charleston Medical Journal and Review*, recommends the following for gastralgia, where an active stimulant and anodyne is required:

℞.—Chloric ether, ℥ss.; bicarb. sodæ, ʒiss.; morphine, gr. iij.; camphor water, ℥iv. Dose, a tablespoonful one, two, or three hours apart until relief is obtained.

*Prurigo Ferox.*—A good prescription for this is said to be Hebras' modification of Wilkinson's ointment, which is composed of the following ingredients: ℞. Suphuris sublim., olei. fagi, aa ℥vj; cretæ preparatæ, ℥iv.; sapo viridis, axunge, aa, libram j. M.—*Amer. Jour. Syph. & Dermat.*

Prof. W. W. Seely (*Clinic*) thinks that facial paralysis may occur from simple congestion or pressure on or around the facial nerve, in inflammations of the middle ear, without any bone involvement; and advises examination of the ear in all cases of paralyzed facial.

*Lotion for Fetid Feet.*—Permanganate of potash, fifteen parts; distilled water, one thousand parts. The feet are to be washed twice a day with the lotion, then to be carefully dried and powdered with potato starch or lycopodium.—*Clinic.*—*Union Medicale.*

---

